# Subjective evaluation of experimental dyspnoea – Effects of isocapnia and repeated exposure

**DOI:** 10.1016/j.resp.2014.12.019

**Published:** 2015-03

**Authors:** Anja Hayen, Mari Herigstad, Katja Wiech, Kyle T.S. Pattinson

**Affiliations:** Nuffield Department of Clinical Neurosciences, University of Oxford, John Radcliffe Hospital, University of Oxford, Oxford OX3 9DU, UK

**Keywords:** Dyspnoea, FMRI, Habituation, Isocapnia, Perception, Respiratory loading

## Abstract

Resistive respiratory loading is an established stimulus for the induction of experimental dyspnoea. In comparison to unloaded breathing, resistive loaded breathing alters end-tidal CO_2_ (P_ET_CO_2_), which has independent physiological effects (e.g. upon cerebral blood flow). We investigated the subjective effects of resistive loaded breathing with stabilized P_ET_CO_2_ (isocapnia) during manual control of inspired gases on varying baseline levels of mild hypercapnia (increased P_ET_CO_2_). Furthermore, to investigate whether perceptual habituation to dyspnoea stimuli occurs, the study was repeated over four experimental sessions. Isocapnic hypercapnia did not affect dyspnoea unpleasantness during resistive loading. A post hoc analysis revealed a small increase of respiratory unpleasantness during unloaded breathing at +0.6 kPa, the level that reliably induced isocapnia. We did not observe perceptual habituation over the four sessions. We conclude that isocapnic respiratory loading allows stable induction of respiratory unpleasantness, making it a good stimulus for multi-session studies of dyspnoea.

## Highlights

• Functional neuroimaging is poised to understand brain processing of dyspnoea.
• Experimental dyspnoea alters PaCO_2_, which confounds FMRI contrast.
• Experimentally stabilizing CO_2_ had minimal effects on perception of respiratory loads.
• No perceptual habituation to resistive loads occurred over four experimental sessions.

## 1 Introduction

Dyspnoea causes tremendous suffering in millions of patients around the world, yet remains poorly understood and is often refractory to treatment (Parshall et al., 2012). Experimentally induced dyspnoea enables its investigation under carefully controlled conditions without the confounds of disease or medications. It is usually achieved by the application of controlled respiratory challenges to healthy research participants. For example, respiratory challenges may be combined with simultaneous functional magnetic resonance imaging (FMRI) in the brain (Herigstad et al., 2011; Pattinson and Johnson, 2014). Respiratory challenges also have direct physiological effects (e.g. on intrathoracic pressure (Hayen et al., 2012)) that may be of interest to some researchers.

Resistive respiratory loading increases variability of partial pressure of arterial and thus end-tidal CO_2_ (P_ET_CO_2_) and may increases or decrease mean P_ET_CO_2_ dependent on the individual's response (Gorman et al., 1999; Hornstein and Ledsome, 1984; McKenzie et al., 1997; Rohrbach et al., 2003; von Leupoldt et al., 2008). Altered arterial CO_2_ is well known to have its own physiological effects, e.g. upon respiratory drive and upon the cardiovascular system (blood pressure, heart rate). Of particular interest in neuroimaging are the profound effects of altered P_ET_CO_2_ on the blood oxygen level dependent (BOLD) response, the source of image contrast in FMRI (Cohen et al., 2002; Wise et al., 2004), which could confound an FMRI study of dyspnoea.

Isocapnia is the term used to describe the maintenance of constant P_ET_CO_2_ by experimenter intervention. It can be used to counteract stimulus-induced CO_2_ effects (Wise et al., 2007). Isocapnia is usually achieved by adding supplemental CO_2_ to the inspired gas mixture (resulting in mild hypercapnia), then P_ET_CO_2_ is fine-tuned on a breath-by-breath basis by adjusting the amount of inspired CO_2_ in the inspired gas mixture depending on the P_ET_CO_2_ recording obtained from the previous breath. This may be done manually or via a computer controlled system (Wise et al., 2007).

Here we investigate whether maintaining isocapnia during respiratory loading could be advantageous for paradigms necessitating alternating on and off periods of dyspnoea, e.g. when investigating the brain mechanisms of dyspnoea with FMRI. Although it is well established that isocapnia would minimize P_ET_CO_2_ variability, it is unknown whether the obligatory hypercapnia would affect the subjective response to respiratory loading. If mild hypercapnia increases unpleasantness, a potential benefit might be a more clinically meaningful stimulus, that could potentially allow a decrease in the intensity of applied respiratory loads (with subsequent reduction in head motion artefact and smaller swings in intrathoracic pressure (Hayen et al., 2012), both contributors to physiological noise). However, baseline hypercapnia may have the deleterious effect of increasing dyspnoea during the unloaded periods (i.e. at baseline).

In naïve participants, training sessions are commonly used to familiarize participants with respiratory stimuli (e.g. (Nishino et al., 2007; O’Donnell et al., 2013)), as the first experience of resistive loading is well known to be associated with a heightened dyspnoea response. It is unknown whether and to what magnitude habituation or sensitization occurs over subsequent sessions of resistive loading. Intervention studies in dyspnoea often compare responses under different conditions (e.g. drug/placebo) over multiple experimental sessions. Habituation (or sensitization) to the stimulus would lead to an order effect, which, if strong enough, might outweigh small intervention-induced differences of interest. Hence, a second aim of this study was to determine the importance of habituation or sensitization of the perceived unpleasantness of experimental dyspnoea stimuli.

In this study, we tested the following hypotheses, all aimed at better understanding experimental dyspnoea for the purposes of the design of FMRI experiments:

• Mild baseline hypercapnia would not increase respiratory unpleasantness significantly, compared to unloaded breathing.
• Combining mild hypercapnia with resistive loading would have an additive effect on dyspnoea unpleasantness.
• There is minimal relevant habituation to dyspnoea as a result of repeated resistive loading over four experimental sessions.

## 2 Materials and methods

### 2.1 Participants

Ten volunteers (aged 23 ± 6 years, 4 females) participated in this study after giving written informed consent in accordance with the Oxfordshire Research Ethics Committee. Participants were healthy right-handed non-smokers with no history of significant psychological, neurological, pulmonary or cardiovascular disease and free from acute illness (e.g. acute upper respiratory tract infection). Participants abstained from heavy meals and physical activity (cycling, exercise) for at least 1 h before each session. The study consisted of five sessions performed on consecutive days. Participants were tested at the same time of day in order to avoid circadian changes in hormone level and cognitive performance throughout the day (Carrier and Monk, 2000), which might affect the perception of dyspnoea (Hayen et al., 2013). To minimize the biasing effects of initial first-time adaptation to a respiratory apparatus, the first session was a training session similar in length and stimulus strength to the subsequent experimental sessions.

### 2.2 Experimental paradigm

#### 2.2.1 General

**Fig. 1:**
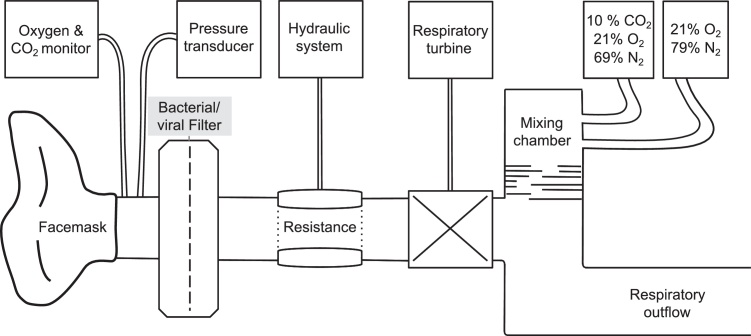
Schematic illustration of custom-built respiratory circuit. A facemask (7450 SeriesV2™ Mask, Hans Rudolph, USA) was connected to two sampling lines measuring respiratory gases and respiratory pressure via polyethylene extension tubing (Vygon SA, Ecouen, France). One sampling line led to a pressure transducer (MP 45, ±50 cmH_2_O, Validyne Corp., Northridge, CA, USA) connected to an amplifier (Pressure transducer indicator, PK Morgan Ltd, Kent, UK) and provided readings of current respiratory pressure at the mouth. Tidal CO_2_ and tidal oxygen were continuously sampled and analyzed using an infrared analyzer with side-stream sampling (Capnomac Ultima, Datex Ohmeda, Helsinki, Finland). The diameter of a 5 cm long section of respiratory tubing is lined with a rubber party balloon connected via non-distensible plastic tubing to a 10 ml syringe filled with water and can be altered remotely to increase respiratory resistance. A turbine (VMM-400, Interface Associates, Aliso Viejo, CA, USA) was used to record inspiratory and expiratory volumes. Medical gases were fed from cylinders and entered a mixing chamber that allowed thorough mixing before inspiration.

**Fig. 2:**
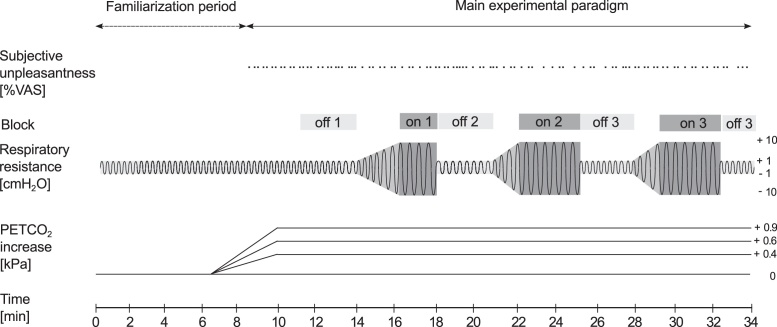
Timeline of experimental session. Each experimental session lasted 34 min. After an adjustment period of 5 min, baseline P_ET_CO_2_ was determined. In the isocapnia conditions, P_ET_CO_2_ was then increased according to condition (+0.4 kPa, +0.6 kPa, +0.9 kPa) and was kept stable at the target P_ET_CO_2_ level. P_ET_CO_2_ was allowed to freely vary during the poikilocapnia condition. Participants rated perceived respiratory unpleasantness at semi-random intervals four times a minute. The strength of respiratory loading applied during the session was determined during the first 2 min of the first respiratory loading block. Three blocks of respiratory loading of 4 min length were administered at 14, 21 and 28 min.

Participants were seated comfortably and were breathing through a custom-built respiratory circuit connected to a facemask (Hans Rudolf; Fig. 1). To this circuit, a mixture of compressed medical air (humidified) was added, in which inspired CO_2_ was adjusted by mixing in varying amounts of a CO_2_-rich gas (10% CO_2_, 21% O_2_, balance nitrogen). To reduce auditory distractions, participants wore standard foam earplugs and headphones (Sony MV300, Sony), through which a constant sound (‘pink noise’) was played at a volume sufficient to mask sounds of the respiratory system (Banzett et al., 1996). The experimental paradigm shown in Fig. 2 was run for all experimental sessions. After a 5-min period in which participants familiarized themselves with breathing on the respiratory circuit, their P_ET_CO_2_ baseline was determined over the following minute. Over the next 5 min, an investigator manually adjusted the inspired CO_2_ to raise P_ET_CO_2_ to the desired amount above baseline. Each participant underwent a poikilocapnia (P_ET_CO_2_ allowed to fluctuate naturally) session and three sessions in which P_ET_CO_2_ was experimentally raised by different amounts from the participant's natural breathing baseline. In the hypercapnia sessions, P_ET_CO_2_ was increased by +0.4 kPa, +0.6 kPa or +0.9 kPa above baseline and the order was randomized.

#### 2.2.2 Resistive respiratory loading

A hydraulic system was used to decrease the diameter of a 5 cm section of respiratory tubing that was lined with a party balloon. Inflating the balloon decreased the diameter of the tube, leading to changes in respiratory resistance during inspiration and expiration. The change in mouth pressure during respiratory loading was titrated to elicit 50% unpleasantness during each individual session in each individual participant. Loads were initially slowly increased over 2 min until subjective ratings indicated 50% VAS for two successive ratings and this mouth pressure was noted as the target level for the rest of the session. A dedicated experimenter maintained mouth pressure constant through breath-by-breath adjustment of inspiratory resistance. During the two successive blocks of respiratory loading during that session, loading was increased over 30 s till the target mouth pressure was reached. Mouth pressures were then kept stable throughout the loading blocks (Fig. 2).

#### 2.2.3 Subjective ratings of respiratory unpleasantness

The perception of breathing-related unpleasantness was recorded at semi-randomized intervals four times a minute during each experimental session, except for the first 11 min of each paradigm (the time during which familiarization with the breathing system took place and P_ET_CO_2_ was adjusted to experimental level). Participants rated their breathing unpleasantness on a horizontal visual analogue scale (VAS) stating the question ‘How unpleasant did your breathing feel?’ with the anchors ‘not unpleasant’ on the left and ‘very unpleasant’ on the right. Behavioural responses were made via a custom-made button box. Standardized instructions relayed the concepts of respiratory intensity and unpleasantness before the training session. An experimenter verbally confirmed participants’ understanding of the concepts of respiratory intensity and unpleasantness before the training session and ensured that participants were able to distinguish both concepts after the training session. Participants were instructed to use the rating scale so that a maximum rating would correlate with the degree of unpleasantness that would cause them to stop the experiment immediately.

#### 2.2.4 Physiological monitoring

Heart rate was measured with a pulse oximeter (Capnomac Ultima, Datex Ohmeda, Helsinki, Finland). All physiological data were sampled at 100 Hz and were logged via a Power1401 using Spike2.7 (CED, Cambridge, UK).

#### 2.2.5 Questionnaires

Participants answered a short pre-study questionnaire immediately before being connected to the respiratory circuit, which contained the following four questions on a horizontal VAS with ‘not at all’ as the left anchor and ‘very’ as the right anchor: “How tense are you feeling? How nervous are you about this session? How bad do you expect this session to be? How relaxed are you feeling right now?”. Immediately after participants were released from the respiratory circuit, they filled in an early version of the Multidimensional Dyspnea Profile (MDP) that measured six sensory qualities of dyspnoea, as well as overall shortness of breath and respiratory unpleasantness (Banzett et al., 2008). After completing the questionnaire, participants were verbally asked “How did your breathing feel during the experiment?”. The experimenter recorded the answers participants gave and prompted for additional information once participants had completed their assessment. Participants were fully debriefed after completion of the experiment.

### 2.3 Data analysis

#### 2.3.1 Preprocessing

Respiratory data (mouth pressure and P_ET_CO_2_), heart rate and unpleasantness ratings were exported to MatLab (MathWorks Inc., Natick, MA, USA) and analyzed on a breath-by-breath basis using custom written scripts. Data were averaged over resistance and non-resistance blocks (excluding the first 120 s of the first block and the first 30 s of the remaining resistance blocks whilst pressure was adjusted to target level). All statistical analyses were performed using the SPSS statistical package version 21 (SPSS Inc., Chicago, IL). Prior to analysis, descriptive statistics were calculated for all variables to ensure the correct choice of test. For all analyses, two-tailed testing at *p* < 0.05 was used to determine statistical significance.

#### 2.3.2 Isocapnia

Fluctuations in P_ET_CO_2_ and their reduction by the isocapnia manipulation were assessed by computing the temporal coefficient of variation (CV) by taking the temporal standard deviation (SD) for each breath, dividing it by the mean and multiplying it by 100 to receive a result in %. A 2 (resistance) × 4 (CO_2_ condition) repeated measures ANOVA was performed to test for differences in the coefficient of variation between CO_2_ conditions. Post hoc tests were based on estimated marginal means and were corrected for multiple comparisons with Bonferroni corrections.

#### 2.3.3 Effects of hypercapnia on unpleasantness

A 2 (resistance) × 4 (P_ET_CO_2_ condition) repeated measures ANOVA was performed to test for differences in unpleasantness ratings. To establish potential effects of hypercapnia on baseline unpleasantness for the lowest potent isocapnia manipulation post hoc that compared non-resistance blocks during poikilocapnia with non-resistance blocks during +0.6 kPa hypercapnia. Additional 2 × 4 repeated measures ANOVAs were performed with the means of mouth pressure amplitude and heart rate.

#### 2.3.4 Habituation

A 2 (resistance) × 3 (block) × 4 (session) repeated measures ANOVA was performed to investigate potential changes in unpleasantness ratings within or between experimental sessions. The same test was performed to check for stability of mouth pressure amplitude and heart rate over time.

*MDP*: One related-samples Friedman's two-way analysis of variance by ranks was performed for each dimension of the MDP comparing all four experimental conditions (df = 3). Due to the exploratory nature of this analysis and its primary aim of highlighting potential changes in specific dimensions in order to focus future analyses, no corrections for multiple comparisons were applied.

#### 2.3.5 Qualitative data

Qualitative data were entered into NVivo qualitative data analysis software (QSR International Pty Ltd. Version 10, 2012). Each statement was coded into a node according to its meaning. Interesting themes emerged from the data and are presented to supplement quantitative data.

## 3 Results

All ten participants tolerated CO_2_ loading and the addition of respiratory loading and completed the experimental protocol. Two participants performed a training session and only three experimental sessions (Table 1).

### 3.1 Isocapnia

**Fig. 3:**
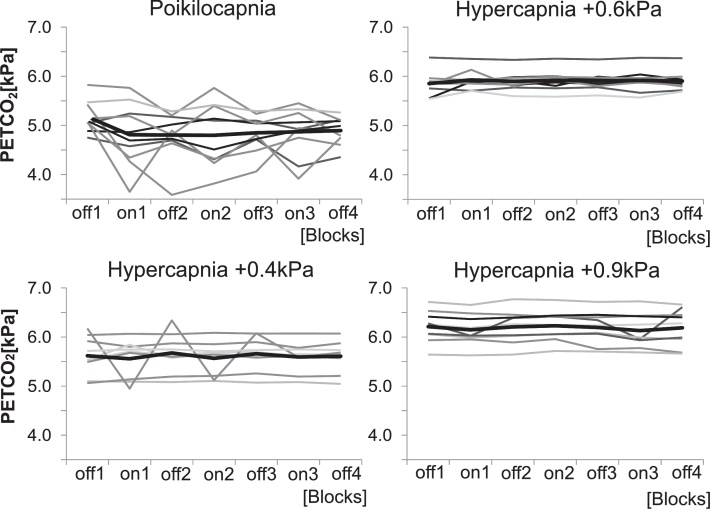
Plot of mean P_ET_CO_2_ averaged over resistance blocks and plotted according to P_ET_CO_2_ session. The black line represents the average of all participants. Individual traces for all participants are plotted in grey shades.

P_ET_CO_2_ fluctuated between blocks with respiratory loading and no respiratory loading during poikilocapnia (Fig. 3). A 2 (resistance) × 4 (CO_2_ condition) repeated measures ANOVA on the coefficient of variability of P_ET_CO_2_ showed a main effect of CO_2_ condition, *F* = (3,5) = 12.99 *p* < .0001. Post hoc tests determined that the P_ET_CO_2_ coefficient of variability decreased with increased hypercapnia (poikilocapnia and 0.4 kPa hypercapnia (*p* = .075), poikilocapnia and 0.6 kPa hypercapnia (*p* = .019), poikilocapnia and 0.9 kPa hypercapnia (*p* = .015)). The ANOVA showed no mean effect for respiratory resistance, *F*(1,7) = 5.09 *p* = .059. An interaction effect of resistance and CO_2_ condition showed a stronger decrease in coefficient of P_ET_CO_2_ variability during resistance periods compared to non-resistance periods with increased hypercapnia, *F*(3,5) = 5.06 *p* = .009 (Fig. 3). There is no significant difference in the coefficient of variability of P_ET_CO_2_ between poikilocapnia and 0.4 kPa hypercapnia (mean difference 2.8, *p* = .131), but a difference between poikilocapnia and 0.6 kPa and 0.9 kPa hypercapnia (poikilocapnia vs. 0.6 kPa: mean difference 4.5 *p* = .033, poikilocapnia vs. 0.9 kPa: mean difference 4.3 *p* = .029). These results suggest 0.6 kPa hypercapnia to be the lowest CO_2_ increase at which manual gas control achieved the desired effect of significantly reducing P_ET_CO_2_ variability compared to poikilocapnia (Fig. 3).

### 3.2 Effects of hypercapnia on respiratory unpleasantness

**Fig. 4:**
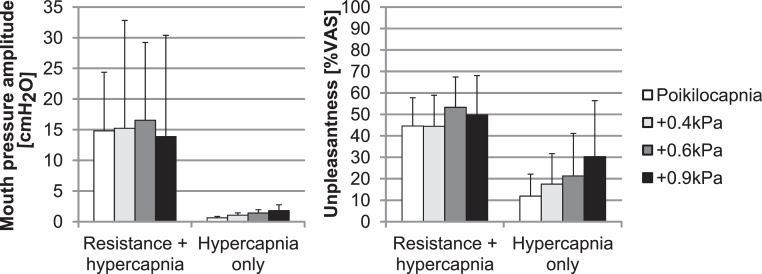
Mouth pressure amplitude and unpleasantness averaged over all participants by experimental condition (error bars depict SD).

A 2 (resistance) × 4 (CO_2_ condition) repeated measures ANOVA performed on unpleasantness ratings showed a main effect of resistance, *F*(1,7) = 45.830 *p* < .0001, no main effect of CO_2_, *F*(3,5) = 1.80 *p* = .178, and no significant interaction *F*(3,5) = .632 *p* = .602 (Fig. 4). The potential increase in unpleasantness caused by the lowest level of isocapnic hypercapnia (+0.6 kPa) compared to poikilocapnia was further investigated, as such an effect could be detrimental during FMRI. A post hoc analysis showed increased respiratory unpleasantness during non-resistance periods during +0.6 kPa hypercapnia compared to non-resistance periods at baseline P_ET_CO_2_ (increase from 12 to 21%VAS, *p* = .020). Results of additional physiological recordings and a sample trace can be found in the Supplementary Material.

### 3.3 Characterization of dyspnoea stimuli

The MDP was used to measure differences in perception during hypercapnia and poikilocapnia. Results are shown in the Supplementary Material.

### 3.4 Habituation of respiratory unpleasantness

**Fig. 5:**
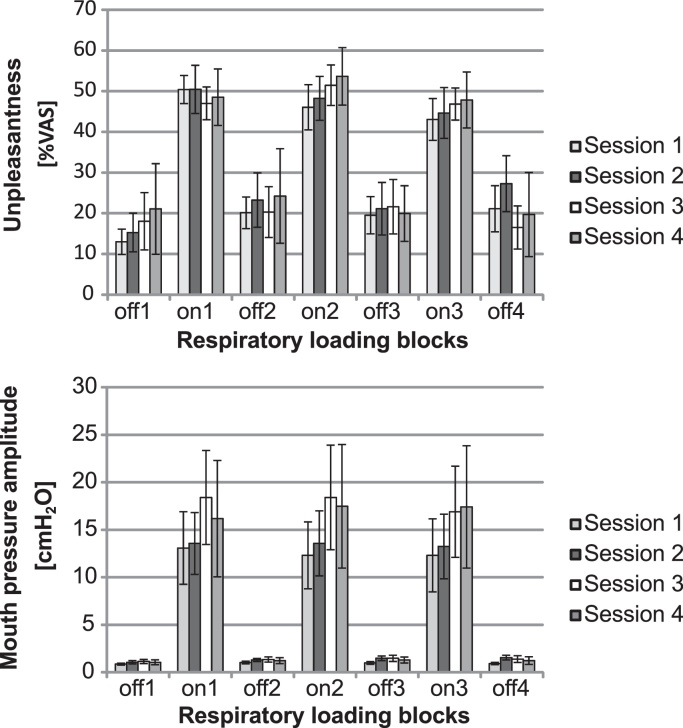
Adaptation within and between sessions. Chronological plot of unpleasantness ratings and mouth pressure amplitudes averaged over all participants (mean ± standard error) during the four no resistance and the three resistance blocks within each session (off1 = first unloaded block, on1 = first respiratory resistance block). Data presented averaged over all poikilocapnia and hypercapnia conditions (counterbalanced) sorted according to session (session 1 = first experimental session after training). * Mean of first non-resistance block is lower than subsequent unloaded blocks at *p* = .05.

Fig. 5 presents average unpleasantness and mouth pressure amplitude for each experimental block of each session.

A 2 (resistance) × 3 (block) × 4 (session) repeated measures ANOVA on average unpleasantness showed a main effect for resistance, *F*(1,7) = 75.53 *p* < .0001, no main effect of block, *F*(2,6) = 2.14 *p* = .154, and no main effect of session, *F*(3,5) = .127 *p* = .943. A significant interaction between block and resistance was obtained, *F*(2,6) = 8.08 *p* = .005, which revealed a trend for unpleasantness ratings during the third block of respiratory loading to be lower compared to the first loading block and for unpleasantness ratings during the third block without respiratory loading to be higher than during the first loading-free block (Fig. 5). These results suggest that no habituation of respiratory unpleasantness occurred within or between sessions.

Mouth pressure, P_ET_CO_2_, P_ET_O_2_ and heart rate remained stable throughout the sessions and the subjective perception of dyspnoea measured with the MDP after each visit remained stable over all four study visits (see Supplementary Material).

### 3.5 Qualitative feedback

The continuous mild hypercapnia used in this study was applied before ratings of respiratory unpleasantness were started, so that it would not be perceived as a change from baseline. During debrief after all sessions, all participants stated that they were not aware that they had received continuous hypercapnia during some of the sessions. In a verbal interview, some participants also mentioned that they found it very difficult to evaluate the unpleasantness of mild respiratory sensations in the lower third of the spectrum. A few participants further mentioned that they were not sure whether the change they noticed was real when it was mild or whether they imagined a change, because they were asked to focus on their breathing and were trying to perceive a change. With regards to side-effects of hypercapnia, two participants reported feeling uncomfortably hot and one participant felt claustrophobic during +0.9 kPa hypercapnia, one participant felt uncomfortably hot during +0.6 kPa hypercapnia.

## 4 Discussion

### 4.1 Main findings

The main findings of this study are:

1. Mild hypercapnia did not amplify respiratory unpleasantness in response to respiratory loading.
2. During unloaded breathing, hypercapnia of +0.6 kPa lead to a small increase in respiratory unpleasantness from 12%VAS to 21%VAS.
3. Unpleasantness of respiratory loading did not habituate within and between sessions.

### 4.2 First study of effects of hypercapnia on unpleasantness during respiratory loading

To our knowledge, this is the first study investigating the effects of hypercapnia on the perception of respiratory loading. Here, we have shown that hypercapnia and respiratory loading did not linearly increase respiratory unpleasantness, but that the same amount of respiratory loading was necessary to induce 50%VAS unpleasantness during all hypercapnia conditions. Hence, adding mild continuous hypercapnia to respiratory loading at the levels used in this study does not provide the additional benefit of reducing the stimulus strength necessary for the induction of moderate dyspnoea unpleasantness, which could reduce physiological confounds of respiratory loading during blood-flow dependent measures such as transcranial Doppler sonography or FMRI.

Future research studies are required to carefully investigate the mechanistic underpinnings of these results, which could be a major milestone in understanding dyspnoea perception. Our findings suggest three potential hypotheses for further investigation:

1. It is possible that intensity and unpleasantness of dyspnoea behave differently with the intensity of dyspnoea increasing more linearly (Nishino et al., 2007) than the unpleasantness, as measured in the current study.
2. Differences in linearity might be due to the different underlying physiological mechanisms by which respiratory loading and hypercapnia cause dyspnoea. It has previously been suggested that the dyspnoea caused by mechanical respiratory loading is mainly caused by activation of lung receptors, whilst hypercapnia mainly causes dyspnoea due to chemoreceptor activation (Lansing et al., 2009). It might be possible that dyspnoea evoked through the same physiological pathway influences sensations in an additive way, whilst dyspnoea that is processed by different afferent pathways is as strong as the most severe dyspnoea perceived.
3. Differences in top-down categorization of respiratory sensations have been shown to override differences in sensory input (Petersen et al., 2014). Hence differential interoceptive categorization of inspiratory loading as a dyspnoea stimulus and hypercapnia as ‘normal breathing’ might account for perceptual stability during respiratory loading.

### 4.3 Effects of hypercapnia on unpleasantness during unloaded breathing

Hypercapnia of +0.6 kPa above resting breathing baseline was necessary for reliable isocapnia (Fig. 3). We observed a small increase of respiratory unpleasantness from 12% (±10) VAS to 21% (±20) VAS when +0.6 kPa hypercapnia was compared with poikilocapnia. Although statistically significant, this effect might be considered small enough to be biologically unimportant. Increased unpleasantness during unloaded breathing is not ideal for the study of dyspnoea with FMRI, as the statistical analysis relies on the comparison of different conditions (e.g. unloaded breathing and respiratory loading) during the same scanning session. Increasing unpleasantness during unloaded breathing would decrease the change between the unloaded condition and respiratory loading on a perceptual and neural level. Despite potentially small perceptual effects, care needs to be taken to not transform isocapnic hypercapnia from a useful manipulation to decrease P_ET_CO_2_ variability into a dyspnoea stimulus in its own right. The benefits of isocapnia for the reliability of physiological recordings (e.g. FMRI signal stability) need to be considered against potentially deleterious subjective effects. We conclude that perceptual ratings during unloaded respiratory periods would be advisable for studies comparing dyspnoea vs. no dyspnoea conditions (e.g. neuroimaging studies) in order to correctly interpret differences between conditions. We also advise that the administration of additional CO_2_ for isocapnia should be kept minimal.

### 4.4 Habituation of respiratory unpleasantness

#### 4.4.1 Within-session habituation

Perceived unpleasantness remained stable over three consecutive 4-min administrations of respiratory loading within one session. This is good news for the investigation of experimental dyspnoea during FMRI, where neural changes are usually obtained by averaging over multiple stimuli. A relatively stable stimulus is therefore necessary to obtain comparable neural responses at the beginning and at the end of the scan and we have shown that respiratory loading fulfils this criterion over the time course of a normal FMRI study.

Unexpectedly, we found that unpleasantness during unloaded periods increased after the initial application of respiratory loading in each session. This effect remained present throughout each experimental session. During poikilocapnia, unpleasantness, mouth pressure and P_ET_CO_2_ were increased after the first respiratory load. Whilst isocapnia eliminated effects of respiratory loading on mouth pressure and P_ET_CO_2_, the difference in unpleasantness remained. This consistent increase in respiratory unpleasantness during unloaded breathing following the initial application of respiratory loading highlights the difficulty of assessing ‘normal breathing’ on a respiratory circuit. Unloaded breathing was rated at 12 (±10) %VAS unpleasantness, which varied throughout the experiment. During the post-study interview, participants stated difficulties determining ‘normal breathing’ while they were on the respiratory circuit and many used a strategy in which they adjusted the rating given for the immediately previous experience by the currently perceived difference in unpleasantness. This is in line with the peak-end effect of dyspnoea discussed by Bogaerts and colleagues (2012), who showed that perceptions at the most intense point (peak) and at the end of dyspnoea application most strongly impacted on immediate recollection of the stimulus. The variability in unpleasantness scores obtained during unloaded breathing and the qualitative interview both highlight the benefits of measuring subjective perception of unloaded breathing throughout the session to aid interpretation of results.

#### 4.4.2 Habituation across sessions

There was no adaptation of respiratory unpleasantness across sessions during loaded or unloaded breathing. This makes respiratory loading a valuable tool for comparing dyspnoea over multiple sessions and allows its use during intervention studies, e.g. pharmacological studies that involve administration of drug and control in separate sessions.

Perception of respiratory unpleasantness was stable in response to repeated respiratory loading. Previous studies have shown varying degrees of perceptual habituation to different respiratory stimuli. No habituation of dyspnoea perception in response to hypercapnia was shown in a study by Bloch-Salisbury and colleagues, who gave hypercapnic challenges to four mechanically ventilated patients with experimentally induced continuous hypercapnia over two weeks (Bloch-Salisbury et al., 1996). Patients retained their original stimulus-response to the load. Whilst perceptual stability was also found during repeated breath holds within one laboratory session (Nishino et al., 1996) and during repeated exercise over 12 sessions in patients with chronic obstructive pulmonary disease (Carrieri-Kohlman et al., 2001), there is evidence of perceptual habituation in response to repeated hypercapnic rebreathing challenges in healthy individuals (Li et al., 2006; Wan et al., 2009). It seems like habituation of dyspnoea perception occurs during short laboratory challenges of hypercapnia, whilst repeated administration of hypercapnia in ventilated patients, respiratory loading, breath holding and exercise show higher perceptual stability. This would need to be investigated within the same study in order to disentangle differences that might originate from differences in study context and exact sensations rated.

Understanding the mechanisms underlying the habituation of sensory and affective responses to respiratory sensations could explain in which contexts and to which stimuli experiential habituation occurs. To our knowledge, potential neuronal mechanisms of habituation to respiratory unpleasantness in response to experimental dyspnoea stimuli have not been investigated, but one study has shown perceptual and neuronal habituation to short inspiratory occlusions (12 blocks) during late compared to early experimental periods using electroencephalography (EEG) (von Leupoldt et al., 2011). This suggests the habituation of neural processing of respiratory sensations as a potential mechanism for reduced respiratory perceptions. Additional clues might be found when investigating the complex interplay between dyspnoea and other cognitive and emotional states. Trait anxiety has been shown to influence the rate of perceptual habituation to dyspnoea stimuli, with higher anxiety resulting in less experiential habituation (Li et al., 2006). A striking additional finding in our study was the large interindividual variation in respiratory unpleasantness. The interindividual differences in perception of dyspnoea in the current study largely outweighed the more subtle differences between hypercapnia conditions and might mask subtle habituation effects of particular groups, which would need to be investigated in future studies.

### 4.5 Benefits of isocapnia when inducing experimental dyspnoea during FMRI

Manual gas control during unloaded breathing and during the application of respiratory loading minimized intra- and interindividual variation of P_ET_CO_2_ (Fig. 3). This has two potential benefits for using respiratory loading to investigate dyspnoea during FMRI. Firstly, isocapnia reduced P_ET_CO_2_ fluctuations within individual sessions compared to poikilocapnia. Controlling P_ET_CO_2_ in this way has the benefit of reducing intraindividual variations in BOLD responsiveness (Harris et al., 2006) and allows the dissociation of the signal of interest from BOLD activity caused by CO_2_ fluctuations. Secondly, isocapnia reduced P_ET_CO_2_ fluctuations between participants. As seen in Fig. 3, application of a respiratory load of 15 cmH_2_O amplitude increased variation in P_ET_CO_2_ within a group of ten participants during poikilocapnia. Previous literature shows hypercapnia or unaltered P_ET_CO_2_ in response to respiratory loading at a group level (Gorman et al., 1999; Hornstein and Ledsome, 1984; Isono et al., 1990; McKenzie et al., 1997; Rohrbach et al., 2003; von Leupoldt et al., 2008), which indicates between-subject variability in respiratory response to respiratory loads. Reducing this variability in P_ET_CO_2_ changes is beneficial for group FMRI studies. Isocapnia can also effectively be maintained through mechanical ventilation (Evans et al., 2002), but this method requires extensive training during which participants learn to consciously suppress respiration in order to allow mechanical ventilation and is hence difficult to administer and confounded by conscious suppression of respiration.

## 5 Conclusion

This study set out to test the applicability of isocapnic respiratory loading to the investigation of dyspnoea during repeated physiological measures that rely on stable P_ET_CO_2_. As expected, isocapnia reduced the considerable P_ET_CO_2_ variability associated with respiratory loading during poikilocapnia, which potentially has considerable benefits for FMRI signal stability. However, there was no advantage of hypercapnia on perception of respiratory loading and a small amount of increased unpleasantness during unloaded breathing could be deleterious if not accounted for. The unpleasantness associated with repeated induction of dyspnoea with respiratory loading remained stable over four sessions, indicating the usability of this model for intervention studies, including multi-session FMRI paradigms. Whilst no one model is presently ideal for the study of breathlessness, we conclude that a carefully titrated combination of continuous mild hypercapnia and respiratory loading (stimulus to induce dyspnoea) will allow the study of the perception of dyspnoea in healthy volunteers during multi-session intervention studies.

## Figures and Tables

**Table 1:** Participant characteristics. F = female, M = male.

Participant number	Age (years)	Sex	Height (cm)	Weight (kg)	Fitness level	Previous respiratory experience	Worst previous breathlessness
1^a^	18	F	167	66	Casual sports	None	After rowing race
2	26	M	190	86	Frequent sports	Mild allergy to dust mites	During intense exercise in hot weather
3	23	F	158	50	Casual sports	Diving	After a long treadmill session
4	22	F	186	77	Frequent sports	Plays wind instrument	After a cross-country race
5	22	M	183	70	No sports	None	During the 1500 m run at school
6	21	M	162	48	No sports	None	None
7^b^	38	M	183	83	No sports	Childhood asthma, diving, respiratory apparatus	Childhood asthma
8	22	F	149	50	Casual sports	None	None
9	18	M	170	65	Casual sports	Diving	After an intense period of running
10	23	M	183	79	Casual sports	None	After a long treadmill session
Median	22		177	68			

a Participant did not complete +0.4 kPa hypercapnia condition.

b Participant did not complete +0.9 kPa hypercapnia condition.
