# Supplementary material for: Subjective evaluation of experimental dyspnoea – Effects of isocapnia and repeated exposure
Source: Respir Physiol Neurobiol. 2015 Mar;208:21–8. doi: 10.1016/j.resp.2014.12.019 (PMC4347539; doi:10.1016/j.resp.2014.12.019)
Supplement: Supplementary file 1 [file mmc1.docx]

SUPPLEMENTARY MATERIAL

**Subjective evaluation of experimental dyspnoea – effects of isocapnia and repeated exposure.**

Anja Hayen*, DPhil; Mari Herigstad*, DPhil; Katja Wiech*, PhD; Kyle T. S. Pattinson*, DPhil FRCA.

*Nuffield Department of Clinical Neurosciences, University of Oxford, John Radcliffe Hospital, University of Oxford, Oxford, OX3 9DU, UK.

**ADDITIONAL RESULTS**

**Table 1:** Mouth pressure amplitude, unpleasantness, PETCO_2_, end-tidal oxygen (PETO_2_) and heart rate averaged over individual experimental blocks for all 10 participants. Blocks are labeled chronologically (first unloaded block = Off 1, first block with respiratory resistance = On 1) within sessions and are presented for poikilocapnia and the three hypercapnia conditions (+0.4kPa, +0.6kPa and +0.9kPa). P = poikilocapnia. CV = coefficient of variation.

| **Variable** | **CO_2_** | **Off 1** | **On 1** | **Off 2** | **On 2** | **Off 3** | **On 3** | **Off 4** | **All off** | **All on** |
| --- | --- | --- | --- | --- | --- | --- | --- | --- | --- | --- |
| Mouth pressure amplitude [cmH_2_O] | P | 0.63 (0.24) | 15.87 (10.11) | 0.67 (0.33) | 14.11 (8.94) | 0.62 (0.22) | 14.43 (10.11) | 0.57 (0.23) | 0.62 (0.23) | 14.80 (9.57) |
|  | +0.4kPa | 0.91 (0.32) | 15.13 (16.66) | 1.11 (0.38) | 15.68 (18.03) | 1.11 (0.49) | 14.89 (18.18) | 1.07 (0.59) | 1.05 (0.38) | 15.23 (17.59) |
|  | +0.6kPa | 1.06* (0.39) | 16.51 (12.46) | 1.35 (0.47) | 16.15 (12.38) | 1.56 (0.67) | 16.99 (13.35) | 1.60 (0.79) | 1.39 (0.56) | 16.55 (12.68) |
|  | +0.9kPa | 1.55* (0.81) | 13.32 (16.42) | 1.81 (0.89) | 15.45 (18.69) | 1.97 (1.00) | 12.83 (14.56) | 1.91 (1.15) | 1.81 (0.94) | 13.87 (16.53) |
| Unplea-santness [%VAS] | P | 9.33 (9.47) | 44.06 (13.37) | 13.01 (10.28) | 46.97 (16.83) | 11.81 (10.86) | 42.81 (14.45) | 13.44 (13.79) | 11.90 (10.24) | 44.61 (13.14) |
|  | +0.4kPa | 14.33 (14.15) | 47.54 (16.93) | 20.15 (19.25) | 45.03 (17.74) | 18.02 (17.98) | 40.85 (13.68) | 17.50 (15.30) | 17.50 (14.19) | 44.48 (14.44) |
|  | +0.6kPa | 14.20 (15.18) | 53.29 (12.16) | 22.16 (22.67) | 55.74 (14.75) | 23.55 (20.50) | 50.87 (17.95) | 25.38 (24.17) | 21.32 (19.81) | 53.30 (14.09) |
|  | +0.9kPa | 29.68 (31.60) | 51.73 (18.48) | 33.06 (27.59) | 50.51 (19.52) | 29.59 (20.16) | 47.10 (19.98) | 28.99 (27.35) | 30.33 (26.04) | 49.78 (18.29) |
| PETCO_2_ [kPa] | P | 5.18 (0.31) | 4.81 (0.64) | 4.80 (0.48) | 4.80 (0.64) | 4.85 (0.37) | 4.87 (0.49) | 4.90 (0.27) | 4.93 (0.28) | 4.83 (0.55) |
|  | +0.4kPa | 5.62 (0.39) | 5.56 (0.39) | 5.68 (0.39) | 5.57 (0.34) | 5.66 (0.34) | 5.59 (0.30) | 5.61 (0.31) | 5.64 (0.35) | 5.57 (0.33) |
|  | +0.6kPa | 5.85 (0.23) | 5.93 (0.19) | 5.90 (0.19) | 5.92 (0.20) | 5.90 (0.19) | 5.92 (0.22) | 5.90 (0.19) | 5.89 (0.19) | 5.92 (0.20) |
|  | +0.9kPa | 6.21 (0.33) | 6.15 (0.31) | 6.21 (0.34) | 6.23 (0.31) | 6.2 (0.33) | 6.13 (0.35) | 6.19 (0.38) | 6.20 (0.34) | 6.17 (0.32) |
| CV PETCO_2_ [%] | P | 3.51(2.34) | 5.86 (4.09) | 3.89 (1.94) | 6.99 (3.65) | 4.84 (1.92) | 6.89 (3.73) | 4.72 (3.40) | 4.24 (1.69) | 6.58 (3.24) |
|  | +0.4kPa | 2.35 (0.95) | 3.27 (2.63) | 2.68 (1.76) | 3.87 (3.51) | 2.01 (0.79) | 3.47 (2.97) | 2.37 (1.68) | 2.35 (1.05) | 3.54 (2.37) |
|  | +0.6kPa | 1.98 (1.69) | 2.08 (1.00) | 2.01 (1.96) | 1.93 (1.02) | 2.14 (0.98) | 2.21 (1.51) | 1.25 (0.86) | 1.85 (1.17) | 2.07 (0.93) |
|  | +0.9kPa | 1.55 (0.75) | 2.08 (1.36) | 1.75 (1.13) | 2.11 (1.04) | 1.84 (1.11) | 2.61 (2.28) | 1.93 (0.92) | 1.77 (0.75) | 2.27 (1.23) |
| PETO_2_ [kPa] | P | 15.07 (0.49) | 15.69 (1.04) | 15.53 (0.88) | 15.51 (0.99) | 15.36 (0.42) | 15.10 (0.89) | 15.17 (0.62) | 15.28 (0.33) | 15.44 (0.71) |
|  | +0.4kPa | 17.17 (0.55) | 16.62 (1.14) | 17.58 (0.40) | 17.01 (0.97) | 17.67 (0.56) | 17.01 (0.94) | 17.44 (0.97) | 17.47 (0.57) | 16.88 (0.98) |
|  | +0.6kPa | 17.70 (0.64) | 17.59 (0.72) | 18.31 (0.53) | 18.06 (0.72) | 18.40 (0.49) | 18.12 (0.70) | 18.41 (0.54) | 18.20 (0.50) | 17.92 (0.67) |
|  | +0.9kPa | 18.10 (0.69) | 17.92 (0.86) | 18.42 (0.46) | 18.12 (0.88) | 18.49 (0.55) | 18.12 (0.81) | 18.43 (0.55) | 18.36 (0.53) | 18.06 (0.84) |
| Heart rate [bpm] | P | 77.57 (11.42) | 79.67 (9.77) | 79.90 (9.95) | 79.86 (10.56) | 80.33 (10.92) | 82.01 (10.26) | 79.60 (11.10) | 79.35 (10.32) | 80.51 (9.95) |
|  | +0.4kPa | 75.82 (8.58) | 79.75 (10.02) | 80.65 (10.60) | 78.76 (8.44) | 78.74 (9.10) | 78.14 (8.83) | 79.15 (8.85) | 78.59 (8.61) | 78.89 (8.54) |
|  | +0.6kPa | 75.49 (10.20) | 75.83 (9.68) | 77.68 (8.38) | 79.55 (10.46) | 80.40 (10.57) | 78.93 (11.04) | 80.46 (11.12) | 78.51 (9.63) | 78.10 (10.17) |
|  | +0.9kPa | 81.92 (12.75) | 80.12 (14.13) | 82.62 (12.31) | 83.22 (13.75) | 82.62 (14.08) | 84.34 (13.82) | 83.76 (13.38) | 82.73 (12.84) | 82.56 (13.59) |

**Table 2:** Basic physiology table. Means for 10 participants over all blocks according to chronological session (1-4). Blocks are labeled chronologically (first unloaded block = Off 1, first block with respiratory resistance = On 1) within sessions and are presented for poikilocapnia and the three hypercapnia conditions (+0.4kPa, +0.6kPa and +0.9kPa). CV = coefficient of variation.

| **Variable** | **Session** | **Off 1** | **On 1** | **Off 2** | **On 2** | **Off 3** | **On 3** | **Off 4** | **All off** | **All on** |
| --- | --- | --- | --- | --- | --- | --- | --- | --- | --- | --- |
| Mouth pressure amplitude [cmH_2_O] | 1 | 0.86 (0.33) | 13.08 (12.08) | 1.03 (0.44) | 12.31 (11.13) | 0.99 (0.47) | 12.31 (12.15) | 0.93 (0.38) | 0.95 (0.38) | 12.57 (11.75) |
|  | 2 | 1.05 (0.54) | 156 (10.27) | 1.30 (0.51) | 13.58 10.82) | 1.48 (0.77) | 13.24 (10.75) | 1.55 (0.78) | 1.34 (0.63) | 13.46 (10.50) |
|  | 3 | 1.14 (0.69) | 18.40 (15.64) | 1.35 (0.87) | 18.41 (17.41) | 1.47 (1.05) | 16.89 (15.18) | 1.39 (1.13) | 1.34 (0.92) | 17.90 (15.93) |
|  | 4 | 1.05 (0.73) | 16.17 (17.32) | 1.23 (0.89) | 17.47 (18.39) | 1.29 (0.89) | 17.41 (18.21) | 1.23 (1.14) | 1.20 (0.88) | 17.02 (17.93) |
| Unpleasant-ness [%VAS] | 1 | 12.99 (9.82) | 50.42 (10.93) | 20.12 (12.29) | 46.06 (17.59) | 19.53 (14.49) | 43.06 (16.28) | 21.12 (17.93) | 18.44 (12.18) | 46.51 (12.71) |
|  | 2 | 15.27 (15.02) | 50.45 (18.73) | 23.24 (21.12) | 48.24 (17.02) | 21.13 (20.42) | 44.65 (19.79) | 27.28 (21.73) | 21.73 (18.31) | 47.78 (17.67) |
|  | 3 | 18.04 (22.29) | 47.02 (12.75) | 20.29 (19.76) | 51.48 (15.73) | 21.61 (21.18) | 46.85 (12.47) | 16.51 (16.77) | 19.11 (19.35) | 48.45 (12.34) |
|  | 4 | 21.06 (31.51) | 48.53 (19.63) | 24.25 (32.86) | 53.65 (19.95) | 19.94 (19.33) | 47.84 (19.45) | 19.69 (29.24) | 21.23 (27.79) | 50.01 (19.01) |
| PETCO_2_ [kPa] | 1 | 5.59 (0.55) | 5.44 (0.70) | 5.35 (0.88) | 5.43 (0.83) | 5.44 (0.76) | 5.53 (0.57) | 5.50 (0.60) | 5.47 (0.67) | 5.47 (0.69) |
|  | 2 | 5.86 (0.35) | 5.71 (0.74) | 5.90 (0.40) | 5.84 (0.60) | 5.89 (0.42) | 5.76 (0.67) | 5.90 (0.48) | 5.89 (0.41) | 5.77 (0.66) |
|  | 3 | 5.73 (0.46) | 5.75 (0.50) | 5.63 (0.48) | 5.69 (0.56) | 5.63 (0.47) | 5.61 (0.59) | 5.57 (0.56) | 5.64 (0.48) | 5.68 (0.55) |
|  | 4 | 5.62 (0.62) | 5.45 (0.73) | 5.63 (0.67) | 5.47 (0.71) | 5.58 (0.69) | 5.55 (0.62) | 5.56 (0.62) | 5.60 (0.64) | 5.49 (0.67) |
| CV PETCO_2_ [%] | 1 | 2.52 (1.75) | 3.52 (2.69) | 2.19 (1.34) | 3.34 (3.16) | 2.93 (2.30) | 3.66 (2.61) | 3.13 (3.03) | 2.69 (1.85) | 3.50 (2.21) |
|  | 2 | 2.15 (1.37) | 3.38 (3.18) | 2.42 (1.66) | 3.40 (3.60) | 2.42 (1.31) | 4.22 (4.64) | 1.50 (1.28) | 2.12 (0.94) | 3.67 (3.67) |
|  | 3 | 1.95 (1.30) | 2.46 (1.83) | 2.68 (2.04) | 4.19 (3.63) | 2.68 (1.41) | 3.77 (2.80) | 3.05 (2.79) | 2.59 (1.57) | 3.47 (2.42) |
|  | 4 | 2.89 (2.36) | 4.15 (4.08) | 3.16 (2.49) | 4.13 (3.18) | 2.94 (2.11) | 3.72 (3.29) | 2.55 (1.79) | 2.89 (1.86) | 4.00 (3.19) |
| PETO_2_ [kPa] | 1 | 16.85 (1.14) | 17.08 (0.85) | 17.40 (1.04) | 17.14 (1.14) | 17.16 (1.32) | 16.58 (1.67) | 16.99 (1.59) | 17.10 (1.20) | 16.93 (1.10) |
|  | 2 | 17.36 (1.26) | 17.68 (0.80) | 17.98 (1.13) | 18.00 (1.00) | 18.09 (1.11) | 18.04 (0.98) | 18.26 (0.95) | 17.92 (1.09) | 17.91 (0.91) |
|  | 3 | 17.07 (1.55) | 16.81 (1.62) | 17.42 (1.53) | 16.96 (1.62) | 17.36 (1.59) | 17.10 (1.48) | 17.26 (1.60) | 17.28 (1.55) | 16.96 (1.56) |
|  | 4 | 16.54 (1.46) | 15.99 (1.25) | 16.81 (1.55) | 16.35 (1.42) | 17.13 (1.52) | 16.40 (1.46) | 16.69 (1.62) | 16.79 (1.51) | 16.25 (1.31) |
| Heart rate [bpm] | 1 | 76.86 (10.53) | 81.02 (9.92) | 81.89 (11.45) | 79.54 (11.32) | 80.39 (11.94) | 81.52 (11.89) | 80.54 (11.91) | 79.92 (10.97) | 80.69 (10.61) |
|  | 2 | 76.50 (12.97) | 77.17 (11.46) | 78.64 (9.50) | 79.69 (11.51) | 79.53 (12.52) | 79.93 (12.28) | 80.49 (12.60) | 78.79 (11.56) | 78.93 (11.57) |
|  | 3 | 80.98 (9.11) | 78.20 (12.12) | 81.65 (11.02) | 80.39 (10.29) | 82.07 (10.44) | 80.69 (10.99) | 81.47 (9.32) | 81.54 (9.59) | 79.76 (10.58) |
|  | 4 | 75.85 (10.82) | 78.74 (10.38) | 77.93 (8.87) | 81.97 (10.99) | 79.96 (9.89) | 81.30 (9.93) | 80.23 (11.37) | 78.49 (9.63) | 80.67 (10.33) |

**Figure 1:** Representative time course of mouth pressure, unpleasantness rating and P_ET_CO_2_ during main part of experiment for one participant during the poikilocapnia session.

Hypercapnia compared to poikilocapnia increased concentration, as measured by the MDP after the experiment, from 37% to 56%VAS (p=.017) and chest tightness from 10% to 37%VAS (p=.028) during application of respiratory loading. Hypercapnia increased the sensation of breathing deeply from 14% to 21%VAS (p=.007) compared to poikilocapnia. There was no difference in any of the other scales of the MDP (Figure 2).

**Figure 2:** Sensory components of respiratory sensations during unloaded breathing (light beige), hypercapnia (average of +0.4kPa, +0.6kPa and +0.9kPa hypercapnia, brown colour), respiratory loading (dark grey) and respiratory loading and hypercapnia (average of +0.4kPa, +0.6kPa and +0.9kPa hypercapnia, light grey). Data presented as box-plots with interquartile ranges. Horizontal lines depict the median. Diamond shapes depict the mean. N=10.

* p<0.05, ** p<0.01 (uncorrected).

The subjective perception of dyspnoea measured with the MDP after each visit remained stable over all four study visits (Figure 3).

**Figure 3:** Subjective perception of dyspnoea measured by the MDP and presented by study visit (visits 1 to 4 after training session). Beige colour: data for unloaded periods, blue colour: data for respiratory loading periods. Data presented as box-plots with interquartile ranges. Horizontal lines depict the median. Diamond shapes depict the mean. N=10.
